# Comparative Analysis of Phytochemical Composition and Antioxidant Activity of Sprouts from Eight Hemp (*Cannabis sativa* L.) Genotypes

**DOI:** 10.3390/foods15142552

**Published:** 2026-07-20

**Authors:** Romina Beleggia, Valentina Giovanniello, Serafino Suriano, Daniela Trono

**Affiliations:** Consiglio per la ricerca in agricoltura e l’analisi dell’economia agraria (CREA), Centro di ricerca cerealicoltura e colture industriali, S.S. 673, Km 25,200, 71122 Foggia, Italy; romina.beleggia@crea.gov.it (R.B.); valentina.giovanniello@crea.gov.it (V.G.); serafino.suriano@crea.gov.it (S.S.)

**Keywords:** industrial hemp, sprouts, phytochemicals, antioxidant activity

## Abstract

Hemp sprouts are emerging as a potential functional food, although their phytochemical composition remains poorly characterized. The study investigated the effect of genotype on growth, phytochemical accumulation, and antioxidant activity in four-day-old sprouts from eight hemp genotypes: Carmagnola Selezionata (CS), Fibrante, Carmaleonte, Codimono, Futura 75, USO 31, Felsinea, and Santhica 27. An integrated approach combining spectrophotometric assays, HPLC, and GC-MS analyses was used. Sprout length showed limited variability across genotypes, whereas biomass accumulation varied markedly, with CS, Fibrante, Felsinea, and Santhica 27 exhibiting the highest fresh and dry weights, and Carmaleonte and Codimono the lowest. Phytosterols (46.3%) and phenolics (33.9%) represented the predominant classes, followed by tocols (17.3%) and carotenoids (2.5%), whereas terpenes occurred only in traces (0.1%) and cannabinoids were not detected in any genotype. Fibrante, Futura 75, and Santhica 27 accumulated comparatively higher levels of several bioactive classes among the genotypes investigated, whereas the remining genotypes displayed more selective enrichment patterns. In particular, Fibrante, Futura 75, and Santhica 27 showed the highest accumulation of phenolics, particularly kaempferol-3-O-rutinoside, apigenin-7-O-glucoside, vitexin, isovitexin, and gallic acid, together with elevated levels of tocopherols and phytosterols. Fibrante also exhibited the highest carotenoid levels, particularly lutein and violaxanthin, and the highest antioxidant activity. CS, Codimono, USO 31, and Felsinea generally showed lower metabolite accumulation, although they maintained a moderate accumulation of specific lipid-soluble antioxidants and terpenes. Carmaleonte displayed the lowest levels across most metabolites. Overall, among the analyzed genotypes, Fibrante, followed by Santhica 27 and Futura 75, exhibited the most favorable combination of phytochemical composition and biomass production, making these genotypes promising candidates for further studies in the field of functional foods.

## 1. Introduction

In recent years, sprouts have attracted increasing interest as food products due to their appealing taste and freshness. They are widely consumed in Asia and have recently gained popularity in Western countries [1]. Compared to mature plants, sprouts are quicker and more sustainable to produce and are richer in nutrients. During germination, large food reserves like polysaccharides, fats, and proteins are broken down into smaller molecules by enzymes to provide energy for the growing sprout [1]. This process makes the sprout more digestible than the mature plant. Sprouts also contain more phytochemicals including phenolic compounds, carotenoids, tocopherols, alkaloids, and terpenoids [1,2], which are known to exert antioxidant, anti-inflammatory, neuroprotective, and immunostimulant effects [3]. Consistently, regular consumption of sprouts has been associated with improved health and a reduced risk of several chronic diseases [4].

Hemp (*Cannabis sativa* L.) is a highly versatile crop belonging to the Cannabaceae family, first domesticated in Central Asia around 12,000 years ago and later spread worldwide [5]. Although its cultivation was restricted in the mid-20th century due to its association with marijuana, industrial hemp has recently regained attention owing to its agronomic, environmental, and industrial value [6]. Hemp is a low-input crop that requires little or no pesticide use and can tolerate heavy metals, making it suitable for phytoremediation [7]. Its fiber and biomass are widely used in textiles, paper, construction materials, bioplastics, and bioenergy [8].

Several hemp tissues are also exploited in agrochemical, cosmetic, and food sectors [9]. Leaves and inflorescences contain numerous phytochemicals, including non-psychoactive cannabinoids such as cannabidiol (CBD) and cannabigerol (CBG), which have attracted interest because of their therapeutic activities [10,11], as well as terpenes and phenolic compounds with antioxidant and anti-inflammatory properties [12,13]. Although hemp inflorescences and leaves are widely used in the preparation of biopesticides, cosmetics, and dietary supplements, their use in the food sector is prohibited or strictly regulated in several countries due to their cannabinoid content. According to the FDA, food products containing CBD are prohibited in interstate commerce in the United States [9]. In the European Union, they are classified as “novel foods,” and the EFSA has very recently published an updated statement establishing a provisional safe intake level of CBD of 0.0275 mg per kilogram of body weight per day (approximately 2 mg per day for a 70 kg individual) [14]. However, to date, none of the applications for CBD-based novel foods have been approved in the EU. In contrast, hemp seeds and their derivatives (e.g., oil, flour, and meal), which are explicitly allowed for food production in many countries [9], are becoming increasingly popular in human nutrition because they are a good source of nutrients, including polyunsaturated fatty acids, essential amino acids, insoluble fiber, and minerals [15], as well as bioactive compounds, such as phenolic compounds, phytosterols, and tocopherols [16]. Although hemp seeds do not accumulate cannabinoids, traces may be found on the seed surface due to contamination from leaves and inflorescences during harvest and postharvest processing [17].

Hemp sprouts are also edible and contain higher levels of phenolic compounds than ungerminated seeds, which translate into markedly enhanced antioxidant activity in sprouts compared with their seed counterparts [18]. Phenolic-rich hemp sprout extracts have shown bioactivity in experimental models, including anti-inflammatory and antimutagenic effects, as well as selective cytotoxicity against cancer cell lines [18,19,20]. Importantly, cannabinoids are absent in hemp sprouts with the seed coat removed, consistent with the lack of glandular trichomes at early developmental stages [19]. These characteristics, combined with their rapid growth cycle and low environmental impact, highlight hemp sprouts as a promising source of bioactive compounds.

The phytochemical composition of sprouts is influenced by both growth conditions and genetic factors, and understanding how these variables affect the accumulation of bioactive compounds is essential for producing sprouts with enhanced functional quality and commercial value. Regarding hemp sprouts, studies have demonstrated that factors such as light exposure, biostimulant application, and germination time significantly affect the accumulation of phenolic compounds and antioxidant activity [18,21,22]. By contrast, the impact of genotype on these traits remains unexplored. The accumulation of phytochemicals may vary greatly by genotype; this can influence the functional properties of hemp sprouts and, consequently, their use as ingredients in value-added food products. In light of this, the objective of the present study was to evaluate the effect of genotype on sprout growth, phytochemical accumulation, and antioxidant activity in eight hemp genotypes and identify genotypes with desirable compositional characteristics for potential use in functional food applications.

## 2. Materials and Methods

### 2.1. Plant Material and Growth Conditions

Seeds from eight hemp cultivars, Carmagnola Selezionata (CS), Fibrante, Carmaleonte, Codimono, Futura 75, USO 31, Felsinea, and Santhica 27, were made available by the Research Centre for Cereal and Industrial Crops of Rovigo, Italy. For each cultivar, 3 g of seeds (~150 seeds) was sown on 22.5 × 22.5 cm plates containing four layers of filter paper moistened with 60 mL sterilized water and incubated in the dark at 22 °C for 4 days. Four independent biological replicates were established, each deriving from a distinct pool of seeds, and germinated separately under identical conditions. Within each replicate, seven plates per genotype were randomly arranged. At harvest, sprouts from plates within the same biological replicate were manually stripped of their seed coats, pooled, and dried in a vacuum oven (Vuototest-Mazzali, Lissone, Italy) at 25 °C for 24 h. The dried sprouts were then ground into powder using a planetary micro mill (Pulverisette 7, Fritsch, Milan, Italy) and stored in a desiccator until analysis.

### 2.2. Determination of Sprout Length and Biomass

Ten sprouts were randomly collected from each plate, and their length was measured from the radicle apex to the tip of the cotyledons. The fresh weight (FW) and the dry weight (DW) of the same sprouts were measured immediately after harvest and after drying, respectively.

### 2.3. Spectrophotometric Assays

#### 2.3.1. Extract Preparation

Fifty milligrams of ground sample was extracted with 1 mL of a methanol:water (80:20 *v*/*v*) solution acidified with 1% HCl, sonicated at room temperature for 30 min, and centrifuged at 10,000× *g* for 10 min at 4 °C. The resulting supernatant was immediately used for the spectrophotometric assays that were carried out using a Perkin Elmer Lambda 650 UV/Vis spectrophotometer (PerkinElmer, Waltham, MA, USA).

#### 2.3.2. Total Phenolic Content

Total phenolic content (TPC) was determined following Beleggia et al. [23] with minor modifications. Two hundred microliters of extract diluted 1:5 was mixed with 750 μL of Folin–Ciocalteu diluted 1:10. After incubation at room temperature for 5 min, the mixture was added with 750 μL of 7% Na_2_CO_3_ and incubated at room temperature for 1 h. Then, the absorbance was measured at 725 nm. Analyses were carried out in three technical replicates, and results were expressed as mg of ferulic acid equivalents (FE) g^−1^ DW.

#### 2.3.3. Total Antioxidant Activity

Total antioxidant activity (TAA) was assessed using both the 2,2′-azinobis(3-ethylbenzothiazoline) 6-sulfonic acid (ABTS) radical scavenging assay and the ferric reducing antioxidant power (FRAP) assay. The ABTS assay was performed following Beleggia et al. [23]. The ABTS^•+^ was generated by mixing 7 mM ABTS with 2.45 mM potassium persulfate (1:1 *v*/*v*). The mixture was incubated at room temperature in the dark for 16 h, and prior to analysis it was diluted with ethanol:water (50:50 *v*/*v*) to reach an absorbance of 0.8 at 734 nm. Then, 20 µL of the extract was added to 2480 μL of the diluted ABTS^•+^ solution, and the absorbance was measured at 734 nm after 10 min. The FRAP assay was carried out according to Berker et al. [24] with minor modifications. FRAP reagent was prepared by mixing 300 mM sodium acetate buffer solution (pH 3.6), 10 mM 2,4,6-tripyridyl-s-triazine, and 20 mM FeCl_3_·6H_2_O in a 10:1:1 ratio and incubating the mixture at 37 °C for 30 min. Twenty-five microliters of the extract were added with 900 μL of FRAP reagent and 1575 μL water. The resulting mixture was incubated for 40 min at room temperature, and then the absorbance was measured at 593 nm. Both analyses were carried out in three technical replicates, and results were expressed as µmol of Trolox equivalents (TE) g^−1^ DW.

### 2.4. HPLC Measurements

#### 2.4.1. Flavonoids and Phenolic Acids

Flavonoids and phenolic acids were analyzed from the same methanolic extract used for the spectrophotometric assays after filtration through a 0.45 µm RC filter following Beleggia et al. [25] with minor modifications. The extract (10 µL) was injected into a 1200 HPLC system equipped with an automatic sampler and a diode-array detector (Agilent Technologies, Waldbronn, Germany) and separated on a reverse-phase C18 column (Kinetex XB-C18 Core Shell, 100 mm × 2.1 mm; 1.7 μm particle size) (Phenomenex, Torrance, CA, USA) maintained at 35 °C. The mobile phase consisted of (A) water with 0.1% formic acid and (B) acetonitrile at a flow rate of 0.5 mL min^−1^, with the following linear gradient program: 5% B for 2 min, from 5% to 30% B for 10 min, from 30% to 55% B for 1 min, from 55% to 70% B for 2 min, isocratic at 70% B for 1 min, and linear gradient from 70% to 5% B for 5 min. Flavonoids and phenolic acids were detected at 280, 320, and 520 nm. Peak identification was carried out by comparing retention times and absorbance spectra with those of authentic standards, and quantification was carried out using calibration curves. Analyses were carried out in three technical replicates, and results were expressed as µg g^−1^ DW.

#### 2.4.2. Tocols

Tocols were extracted and quantified following Beleggia et al. [26]. The ground sample (100 mg) was extracted with 3 mL of acetonitrile, incubated for 30 min, and centrifuged at 3000× *g* for 15 min at 10 °C. The supernatant was evaporated to dryness using a speed vacuum concentrator (Speedvac Jouan RC1022, Thermo Electron Corporation, West Palm Beach, FL, USA), reconstituted in 1 mL of methanol, and filtered through a 0.22 µm PTFE membrane filter (Millipore, Carrigtwohill, Co., Cork, Ireland). The extract (20 μL) was injected into an 1100 HPLC system equipped with a fluorescence detector (Agilent Technologies, Waldbronn, Germany) and separated on a Zorbax SB-C18 column (250 mm × 4.6 mm; 5 μm particle size) (Agilent, Santa Clara, CA, USA) maintained at 30 °C. The mobile phase consisted of acetonitrile/methanol/2-propanol (40:55:5 *v*/*v*/*v*) under isocratic conditions at a flow rate of 0.8 mL min^−1^ and a total run time of 30 min. Tocols were detected at 280 nm excitation wavelength and 320 nm emission wavelength. Peak identification was carried out by comparing retention times and emission spectra with those of authentic standards, and quantification was carried out using calibration curves. Analyses were carried out in three technical replicates, and results were expressed as µg g^−1^ DW.

#### 2.4.3. Carotenoids

Carotenoids were extracted and quantified following Sestili et al. [27]. The ground sample (100 mg) was extracted twice with 2 mL of hexane/acetone (80:20 *v*/*v*) added with 300 µL of 0.1% (*w*/*v*) butylated hydroxytoluene, stirred in the dark for 16 h, and centrifuged at 3400× *g* for 10 min at 4 °C. The two supernatants were pooled, filtered through a 0.45 µm RC filter (Phenomenex, Torrance, CA, USA), and a 1.7 mL aliquot was evaporated to dryness using a speed vacuum concentrator (Speedvac Jouan RC1022, Thermo Electron Corporation, USA). The dry residue was reconstituted in 0.25 mL of methanol and dichloromethane mixture (45:55 *v*/*v*) and 20 µL was injected into a 1200 HPLC system equipped with a diode-array detector (Agilent Technologies, Waldbronn, Germany). Separation was achieved on a YMC Carotenoid column (250 mm × 4.6 mm; 5 µm particle size) (CPS analitica, Milano, Italy) maintained at 35 °C. The mobile phase consisted of methanol and methyl-*tert*-butyl ether (89:11 *v*/*v*) pre-degassed by sonication for 10 min at a constant flow rate of 1.2 mL min^−1^. Carotenoids were detected at 450 nm. Peak identification was carried out by comparing retention times and absorbance spectra with those of authentic standards, and quantification was carried out using calibration curves. Analyses were carried out in three technical replicates, and results were expressed as µg g^−1^ DW.

### 2.5. GC-MS Measurements

#### 2.5.1. Phytosterols

Phytosterols were extracted following Islam et al. [28] with minor modifications. Briefly, 50 mg of sample was weighed into a screw-cap glass tube, and 3 mL of ethanol containing 0.1% (*w*/*v*) ascorbic acid and 50 µL of 5α-cholestane (100 µg mL^−1^, internal standard) were added. The mixture was manually shaken and heated at 85 °C for 5 min in a shaking water bath. Then, 500 µL of 80% (*w*/*v*) KOH was added and the sample was saponified for 10 min under the same conditions. After cooling, 1 mL of pure water and 1.5 mL of hexane were added and vigorously mixed. After centrifugation at 2900× *g* for 5 min at 4 °C, the hexane phase was collected. Extraction was repeated two more times with 1 mL of hexane. A 500 µL aliquot of the combined extracts was dried in the speed vacuum concentrator (Speedvac Jouan RC1022, Thermo Electron Corporation, USA). The dried residue was derivatized with 50 µL of *N*-methyl-*N*-(trimethylsilyl) trifluoroacetamide and 50 µL of pyridine at 60 °C for 30 min, and then cooled and transferred into GC-MS vials. GC-MS analysis was performed using an Agilent 6890A GC coupled to a 7000B Triple Quadrupole MS (Agilent Technologies, Santa Clara, CA, USA). One microliter of extract was injected in splitless mode and separation was achieved on an HP-5ms capillary column (30 m × 0.25 mm; 0.25 µm film thickness) (Agilent, Santa Clara, CA, USA) using the following oven program: 70 °C for 1 min, ramped at 8 °C min^−1^ to 220 °C and maintained for 1 min, and then ramped at 5 °C min^−1^ to 300 °C and maintained for 15 min. The injector and transfer line temperatures were set at 280 °C, whereas the source temperature was set at 240 °C. Helium was used as carrier gas at 1 mL min^−1^. The MS operated in EI mode (70 eV; 50–700 amu). Phytosterols were identified by comparison with authentic standards, NIST11 spectra, and retention indices from a C8–C34 n-alkane series. Semi-quantification was based on peak normalization to the internal standard and sample weight. Analyses were carried out in three technical replicates, and results were expressed as µg g^−1^ DW.

#### 2.5.2. Terpenes and Cannabinoids

Terpenes and cannabinoids were analyzed following Sicignano et al. [29] with minor modifications. The ground sample (200 mg) was spiked with 10 µL pentadecane (11.5 mg mL^−1^, internal standard) and extracted with 5.5 mL hexane using an accelerated solvent extractor (ASE350, Thermo Fisher Scientific, Waltham, MA, USA) at 50 °C and 10 MPa (5 min heating, 15 min static extraction, 1 cycle). The analysis was performed using the same GC-MS system and column used for the analysis of phytosterols. One microliter of extract was injected in splitless mode and separation was achieved using the following oven program: 60 °C for 1 min, ramped at 8 °C min^−1^ to 220 °C and maintained for 1 min, and then ramped at 8 °C min^−1^ to 280 °C and maintained for 25 min, with a post-run at 300 °C for 1 min. The injector and transfer line temperatures were set at 280 °C, whereas the source temperature was set at 240 °C. Helium was used as carrier gas at 1 mL min^−1^. The MS operated in EI mode (70 eV; 50–700 amu). Terpenes and cannabinoids were identified by comparison with authentic standards, NIST11 spectra, and retention indices from a C8–C34 n-alkane series. Semi-quantification was based on peak normalization to the internal standard and sample weight. Analyses were carried out in three technical replicates, and results were expressed as µg g^−1^ DW.

### 2.6. Statistical Analysis

Using JMP software (version 8.0; SAS Institute Inc., Cary, NC, USA), Tukey’s post hoc test (*p* < 0.05) was applied to assess significant differences among group means. Correlation, hierarchical clustering, and heatmap analyses were performed using the MetaboAnalyst 6.0 web platform (https://www.metaboanalyst.ca; accessed 30 April 2026). For these analyses, data were auto-scaled (mean-centered and divided by the standard deviation).

## 3. Results

### 3.1. Sprout Length and Biomass Accumulation Across the Eight Genotypes

Sprout length varied only slightly among genotypes (6.6–8.0 cm), with Fibrante producing the longest sprouts and Carmaleonte the shortest (Table 1). In contrast, sprout FW and DW varied markedly (49.7–89.5 mg and 7.4–12.7 mg, respectively), with the highest values detected in Santhica 27 followed by CS, Fibrante, and Felsinea, and the lowest in Carmaleonte and Codimono. No significant differences were observed in the DW/FW ratio.

### 3.2. Sprout Phytochemical Profile Across the Eight Genotypes

The phytochemical composition of the eight genotypes was characterized using an integrated analytical approach. Phenolic compounds, tocols, and carotenoids were identified and quantified by HPLC, phytosterols, terpenes, and cannabinoids were determined by GC-MS, and spectrophotometric assays were used to measure TPC and TAA. For each metabolite class, total content was calculated as the sum of the individual compounds detected chromatographically for that class. Among the detected metabolites, phytosterols (46.3%) and phenolic compounds (33.9%) dominated the phytochemical profile, followed by tocols (17.3%) and carotenoids (2.5%), while terpenes were present only in trace amounts (0.1%). Cannabinoids were not detected in any of the genotypes investigated, as no chromatographic peaks fulfilling the method identification criteria were observed.

#### 3.2.1. Flavonoid and Phenolic Acid Content and Composition

**Figure 1:**
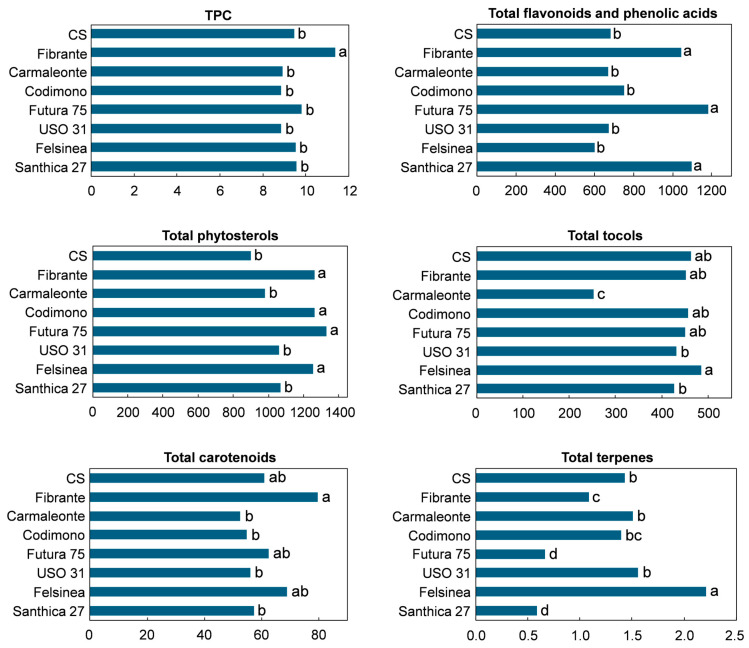
Sprout content of the different metabolite classes across the eight genotypes investigated. Bars represent mean values expressed as mg ferulic acid equivalents g^−1^ dry weight (DW) for TPC and as μg g^−1^ DW for the metabolite classes detected chromatographically. For each metabolite class, total content was calculated as the sum of the individual compounds detected for that class. In each graph, different lower-case letters above the bars indicate significant differences among genotypes according to Tukey’s HSD test (*p* < 0.05).

TPC exhibited limited variability, with only Fibrante showing a significantly higher value than the other genotypes (11.36 vs. 8.83–9.78 mg FE g^−1^ DW). In contrast, HPLC analysis distinguished two groups: Fibrante, Futura 75, and Santhica 27, which showed higher total flavonoid and phenolic acid content (1038.78–1176.77 μg g^−1^ DW), and CS, Carmaleonte, Codimono, USO 31, and Felsinea, with lower content (598.59–746.74 μg g^−1^ DW) (Figure 1).

**Figure 2:**
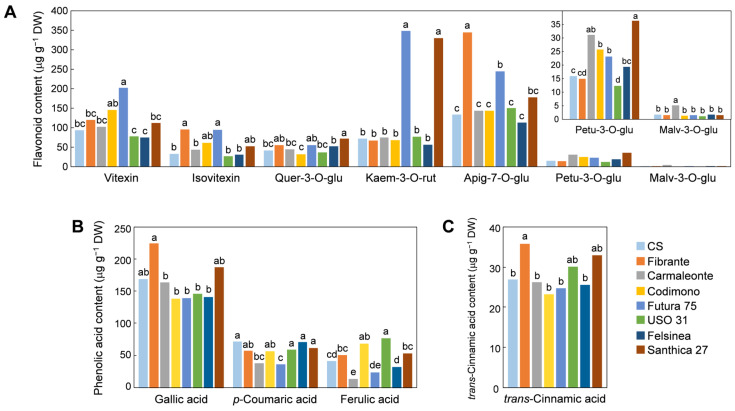
Sprout flavonoid (**A**), phenolic acid (**B**), and *trans*-cinnamic acid (**C**) content across the eight genotypes investigated. Quer-3-O-glu, Quercetin-3-O-glucoside; Kaem-3-O-rut, Kaempferol-3-O-rutinoside; Apig-7-O-glu, Apigenin-7-O-glucoside; Petu-3-O-glu, Petunidin-3-O-glucoside; Malv-3-O-glu, Malvidin-3-O-glucoside. For each compound, different lower-case letters above the bars indicate significant differences among genotypes according to Tukey’s HSD test (*p* < 0.05).

Given the non-selective nature of the TPC assay, the higher values obtained by the Folin–Ciocalteu assay compared with the sum of phenolic compounds detected by HPLC likely reflect the contribution of phenolic compounds other than flavonoids and phenolic acids, together with other reducing non-phenolic compounds, which are less influenced by genotypic variability. Flavonoids and phenolic acids accounted for 67.8% and 32.2% of total phenolics, respectively, with vitexin, kaempferol-3-O-rutinoside, apigenin-7-O-glucoside, and gallic acid as the most abundant compounds (14.0%, 16.5%, 21.9%, and 19.8%, respectively). The higher total content of Fibrante, Futura 75, and Santhica 27 primarily reflected higher levels of kaempferol-3-O-rutinoside, apigenin-7-O-glucoside, and gallic acid (up to 349.38, 345.86, and 225.33 μg g^−1^ DW, respectively) compared to the remaining genotypes (Figure 2). In addition, Futura 75 exhibited the highest accumulation of vitexin (203.22 μg g^−1^ DW) and, together with Fibrante, of isovitexin (95.30 and 95.83 μg g^−1^ DW, respectively), whereas Santhica 27 had the highest levels of quercetin-3-O-glucoside and petunidin-3-O-glucoside (72.42 and 36.53 μg g^−1^ DW, respectively). Fibrante also presented the highest level of *trans*-cinnamic acid (35.94 μg g^−1^ DW). In contrast, CS, Carmaleonte, Codimono, USO 31, and Felsinea generally showed lower accumulation of flavonoids and phenolic acids, with a few exceptions concerning low-abundance metabolites, such as *p*-coumaric acid and ferulic acid.

#### 3.2.2. Phytosterol Content and Composition

**Figure 3:**
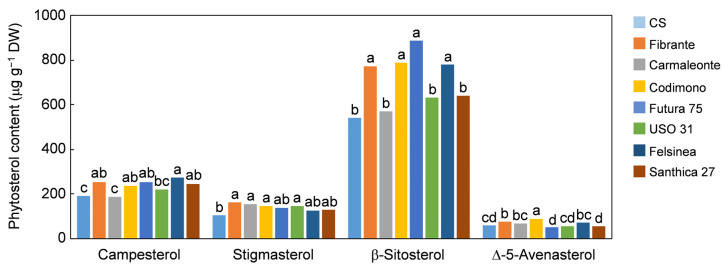
Sprout phytosterol content across the eight genotypes investigated. For each compound, different lower-case letters above the bars indicate significant differences among genotypes according to Tukey’s HSD test (*p* < 0.05).

As regards total phytosterol content, all genotypes exhibited relatively high levels. However, two groups can be distinguished: one including Fibrante, Codimono, Futura 75, and Felsinea, characterized by higher values (1252.53–1327.79 µg g^−1^ DW), and another including CS, Carmaleonte, USO 31, and Santhica 27, which displayed lower values (897.69–1067.58 µg g^−1^ DW) (Figure 1). Among individual phytosterols, β-sitosterol was the most abundant (61.8%), followed by campesterol (20.4%), and stigmasterol (12.1%), while Δ-5-avenasterol was the least abundant (5.8%) (Figure 3). Accordingly, the trend in β-sitosterol resembled that of total phytosterols, with higher levels detected in Fibrante, Codimono, Futura 75, and Felsinea compared to the other genotypes (771.01–887.58 µg g^−1^ DW vs. 542.53–642.56 µg g^−1^ DW) (Figure 3). Campesterol also showed significant genotypic variation, with the highest value detected in Felsinea (273.26 μg g^−1^ DW) and the lowest in CS and Carmaleonte (190.67 and 185.17 μg g^−1^ DW, respectively). Stigmasterol did not vary significantly among genotypes, except for CS, which displayed a lower level than the other genotypes (104.09 vs. 125.9–163.78 µg g^−1^ DW). Codimono exhibited the highest Δ-5-avenasterol content (89.13 vs. 51.79–77.27 µg g^−1^ DW).

#### 3.2.3. Tocol Content and Composition

**Figure 4:**
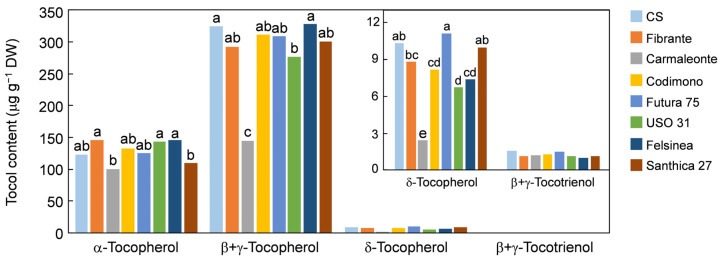
Sprout tocol content across the eight genotypes investigated. For each compound, different lower-case letters above the bars indicate significant differences among genotypes according to Tukey’s HSD test (*p* < 0.05).

With the exception of Carmaleonte, which exhibited the lowest total tocol content (250.66 µg g^−1^ DW), the remaining genotypes exhibited comparable contents, ranging from 424.18 µg g^−1^ DW in Santhica 27 to 484.05 µg g^−1^ DW in Felsinea (Figure 1). This pattern was mainly driven by β+γ-tocopherol, which represented the dominant fraction in this class of phytochemicals, accounting for 67.5% of the total tocols detected, followed by α-tocopherol (30.4%), δ-tocopherol (1.9%), and β+γ-tocotrienol (0.3%) (Figure 4).

Indeed, β+γ-tocopherol content was relatively consistent across genotypes (277.50–329.09 µg g^−1^ DW) except in Carmaleonte, where the content (146.10 µg g^−1^ DW) was approximately half of the other genotypes (Figure 4). α-Tocopherol content showed limited variability, with Fibrante, USO 31 and Felsinea exhibiting the highest values (144.99–146.85 µg g^−1^ DW), and Carmaleonte and Santhica 27 the lowest (101.02 and 110.92 µg g^−1^ DW, respectively). A more pronounced variability characterized the δ-tocopherol content, which reached its highest values in CS, Futura 75 and Santhica 27 (9.99–11.14 µg g^−1^ DW), and the lowest in Carmaleonte (2.41 µg g^−1^ DW). No significant differences were detected in β+γ-tocotrienol content.

#### 3.2.4. Carotenoid Content and Composition

**Figure 5:**
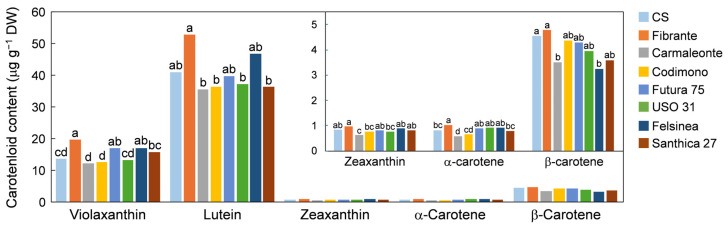
Sprout carotenoid content across the eight genotypes investigated. For each compound, different lower-case letters above the bars indicate significant differences among genotypes according to Tukey’s HSD test (*p* < 0.05).

The highest total carotenoid content was detected in Fibrante (79.45 µg g^−1^ DW) followed by CS, Futura 75, and Felsinea (60.79–68.65 µg g^−1^ DW), whereas Carmaleonte, Codimono, USO 31, and Santhica 27 exhibited the lowest values (52.41–57.42 µg g^−1^ DW) (Figure 1). As for the individual carotenoids, lutein was the predominant compound, accounting for 66.1% of total carotenoids, followed by violaxanthin (24.6%), β-carotene (6.6%), and minor contributions from zeaxanthin and α-carotene (1.3% each) (Figure 5). Fibrante exhibited the highest content for all the individual carotenoids, reaching 19.79 µg g^−1^ DW for violaxanthin, 52.90 µg g^−1^ DW for lutein, 0.96 µg g^−1^ DW for zeaxanthin, 1.03 µg g^−1^ DW for α-carotene, and 4.77 µg g^−1^ DW for β-carotene (Figure 5). Futura 75 and Felsinea also displayed relatively high levels of violaxanthin (16.96 and 16.99 µg g^−1^ DW, respectively) and, together with CS, of lutein (39.61–46.69 µg g^−1^ DW). In contrast, Carmaleonte, Codimono, USO 31, and Santhica 27 exhibited generally lower carotenoid accumulation, especially with respect to violaxanthin (12.18–15.86 µg g^−1^ DW) and lutein (35.50–37.15 µg g^−1^ DW).

#### 3.2.5. Terpene Content and Composition

Terpenes were detected at very low levels in hemp sprouts and belonged exclusively to the sesquiterpene class (Figure 1 and Table 2). The genotypes differed markedly in both profile complexity and quantitative accumulation. Felsinea exhibited the highest total terpene content (2.21 µg g^−1^ DW) and a complex profile, with relatively high levels of β-caryophyllene (0.37 µg g^−1^ DW), α-humulene (0.44 µg g^−1^ DW), alloaromadendrene oxide (0.73 µg g^−1^ DW), and α-bisabolol (0.66 µg g^−1^ DW), whereas β-guaiene and δ-cadinene were absent. In contrast, Futura 75 and Santhica 27 exhibited the lowest total terpene content (0.66 and 0.58 µg g^−1^ DW, respectively) and the simplest profiles characterized by low β-caryophyllene and α-humulene content (0.27–0.33 µg g^−1^ DW) and absence of other terpenes. The remaining genotypes showed intermediate total terpene content (1.08–1.55 µg g^−1^ DW) and profile complexity, with moderate β-caryophyllene and α-humulene content (0.25–0.40 µg g^−1^ DW) and a different distribution of the other minor compounds.

### 3.3. Sprout Total Antioxidant Activity Across the Eight Genotypes

TAA exhibited significant differences among genotypes when evaluated both as radical scavenging activity (ABTS assay) and as reducing power (FRAP assay) (Table 3). For the ABTS assay, Fibrante showed the highest value (10.49 μmol Trolox g^−1^ DW) significantly exceeding Carmaleonte, USO 31, and Felsinea (8.15–8.51 μmol Trolox g^−1^ DW), while intermediate values were detected in the remaining genotypes (8.66–9.76 μmol Trolox g^−1^ DW). A similar trend was observed for the FRAP assay, with Fibrante showing the highest value (18.51 μmol Trolox g^−1^ DW), significantly higher than Carmaleonte, Codimono, and USO 31 (13.86–14.72 μmol Trolox g^−1^ DW), while the other genotypes displayed intermediate values (15.83–16.71 μmol Trolox g^−1^ DW).

**Figure 6:**
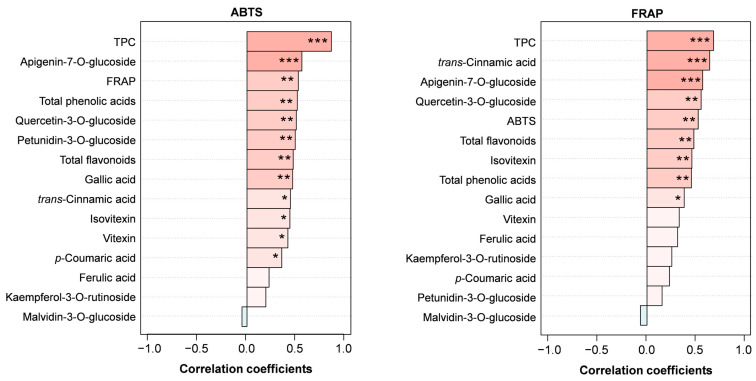
Pearson correlation coefficients between total antioxidant activity measured by the ABTS and FRAP assays and TPC, flavonoids, phenolic acids, and *trans*-cinnamic acid detected by HPLC. Pink bars represent positive correlations, while blue bars represent negative correlations. *, **, and *** are significant at 0.05, 0.01, and 0.001 levels, respectively.

Correlation analysis revealed that both assays showed highly positive correlations with TPC, and several individual phenolic compounds (Figure 6). TPC showed the strongest positive correlation with both ABTS and FRAP (r = 0.857 and 0.678, respectively), whereas, among the individual phenolics, the highest positive correlation was observed for apigenin-7-O-glucoside (ABTS, r = 0.557; FRAP, r = 0.567). Quercetin-3-O-glucoside, total phenolic acids, total flavonoids, gallic acid, and isovitexin also showed a significant positive correlation with both ABTS and FRAP (r = 0.0.379–0.551). *trans*-Cinnamic acid was significantly associated with both assays, although the relationship was significantly stronger with FRAP (r = 0.638) than with ABTS (r = 0.442). Compared to FRAP, ABTS displayed a broader distribution of correlations, which also included petunidin-3-O-glucoside, vitexin, and *p*-coumaric acid (r = 0.353–0.490). A positive correlation was also observed between ABTS and FRAP (r = 0.521), which confirmed that the two assays share reliance on electron transfer mechanisms, while still reflecting partially different compound sensitivities [30].

### 3.4. Hierarchical Clustering and Heatmap Analysis

**Figure 7:**
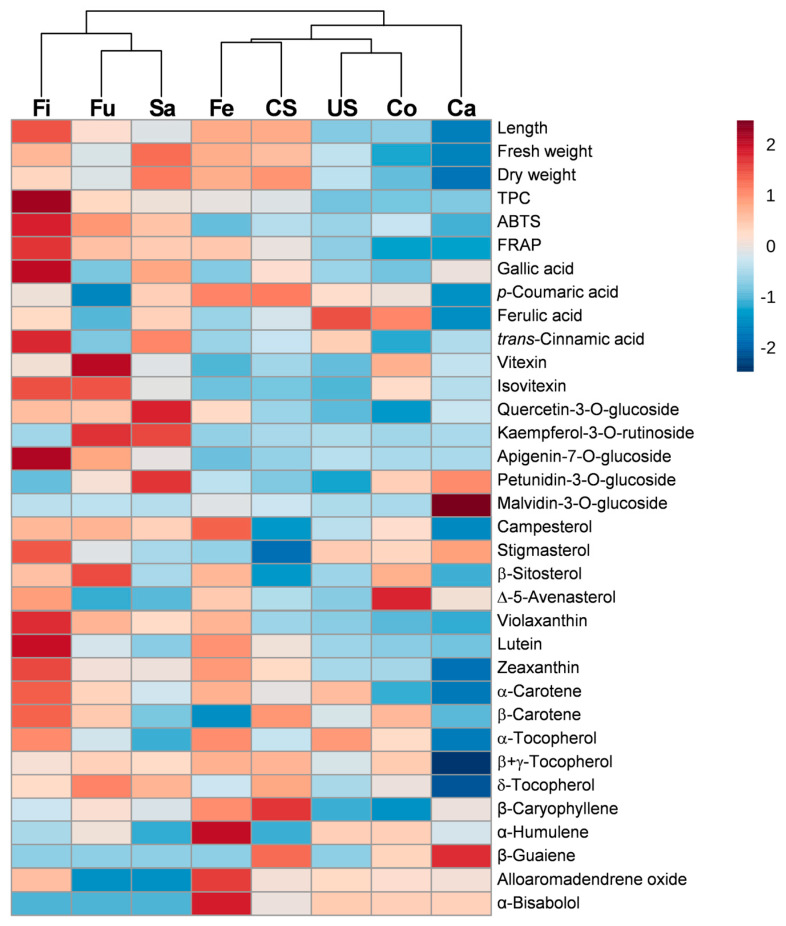
Hierarchical clustering and heatmap analysis of significantly different traits in sprouts of the eight genotypes. Sa, Santhica 27; Fi, Fibrante, Fu, Futura 75; Fe, Felsinea; CS, Carmagnola Selezionata; US, USO 31; Co, Codimono; Ca, Carmaleonte.

Hierarchical clustering analysis distinguished two main clusters: the first grouping Fibrante, Futura 75, and Santhica 27, and the second including Felsinea, CS, USO 31, Codimono, and Carmaleonte (Figure 7). As shown by the heatmap, genotypes in the first cluster exhibited higher TPC, TAA, and a broader accumulation of several bioactive compounds compared to the second cluster. In addition, they also exhibited favorable growth-related traits. Within this cluster, Fibrante was distinguished from the other two genotypes by a more consistent and uniformly elevated profile across most evaluated traits, whereas Santhica 27 and Futura 75 showed greater heterogeneity, with some compounds, especially phenolic compounds and tocols, reaching levels comparable to those of Fibrante and others showing more moderate accumulation. Within the second cluster, three subclusters were distinguished. The first included Felsinea and CS, which exhibited high values of growth-related traits, along with moderate TAA and selective enrichment in certain metabolites, particularly certain carotenoids and tocols; Felsinea also showed high phytosterol accumulation. USO 31 and Codimono formed the second subcluster and displayed lower growth performance and generally low-to-moderate values across most traits. Carmaleonte stood out as a distinct subcluster characterized by the lowest overall values for most of the evaluated traits.

## 4. Discussion

### 4.1. Effect of Genotype on Sprout Growth and Biomass Accumulation

Genotype had a greater influence on biomass accumulation than on sprout elongation, with the highest biomass accumulation detected in CS, Fibrante, Felsinea, and Santhica 27. The DW/FW ratio remained relatively stable, thus suggesting that genotype predominantly influenced total biomass accumulation rather than biomass composition or tissue hydration. From a nutritional point of view, the DW/FW ratio is particularly relevant since it provides indirect information on nutrient density. The values obtained in the present study (0.14–0.16) are comparable to those reported for radish and broccoli sprouts (0.16) and are higher than those of other commonly consumed sprouts, such as black medick, sunflower, leek, beetroot, and mung bean sprouts (0.047–0.086) [31]. In terms of practical application, the higher biomass accumulation observed in the CS, Fibrante, Felsinea, and Santhica 27 genotypes is clearly advantageous for commercial sprout production because it permits higher yields without compromising nutrient density, as shown by the comparable DW/FW ratios among the various genotypes. However, determining a sprout genotype’s potential for food applications requires more than just biomass production. To identify the genotypes most appropriate for the production of functional foods, the agronomic advantages linked to high biomass accumulation should be taken into account in addition to phytochemical composition and antioxidant activity.

### 4.2. Phytochemicals and Antioxidant Activity in Hemp Sprouts

The present study adopted an integrated metabolomic approach to comprehensively characterize the phytochemical profile of hemp sprouts from different genotypes. These data expand the currently limited information on genotype-dependent phytochemical variability in this species. In contrast, previous studies on hemp sprouts have mainly focused on phenolic compounds, often reported only as TPC, with limited information on individual phenolic acids, flavonoids, cannabinoids, and their variation across genotypes [18,20,22,32,33,34].

Phenolic compounds represented one of the most abundant classes of phytochemicals in the sprouts of the investigated hemp genotypes. TPC in sprouts was significantly higher than that previously reported in seeds of the same genotypes (8.3–11.4 mg FE g^−1^ DW vs. 3.9–4.5 mg FE g^−1^ DW) [35], confirming that germination promotes the accumulation of phenolic compounds, as reported in other species [36]. This increase is probably due to the de novo biosynthesis of phenolic compounds through the phenylpropanoid pathway and the enzymatic release of bound phenolics, which may contribute to counteracting the oxidative stress associated with the activation of intense metabolic activity during germination [37]. Although both flavonoids and phenolic acids have previously been identified in hemp sprouts, their reported concentrations vary greatly among studies. The levels observed here are comparable to those of AlJuhaimi et al. [33] but differ from the much higher values reported by Aloo et al. [32] and the considerably lower levels observed by Galanty et al. [20] in dark-grown sprouts. Such variability probably reflects differences in genotype, germination and postharvest storage conditions, as well as extraction protocols and analysis approaches, all of which can significantly affect both the accumulation and quantification of phenolic compounds. Correlation analysis revealed that TPC and several individual phenolic compounds were positively associated with the total antioxidant activity measured by both the ABTS and FRAP assays, thus suggesting that the overall phenolic pool, rather than a single metabolite, largely determines the antioxidant properties of hemp sprout extracts.

Cannabinoids were not detected in any of the genotypes investigated, in agreement with the findings of Werz et al. [19] for sprouts from which the seed coat had been removed. Conversely, trace amounts of cannabinoids (up to 46 μg g^−1^) have been reported in sprouts retaining the seed coat [32,34], thus supporting the hypothesis that these compounds originate from contamination of the seed surface with material from leaves or inflorescences during harvest and postharvest processing, rather than from de novo biosynthesis by the sprouts [17]. Similarly, terpene accumulation was very low or even undetectable in some genotypes. This is likely because, in this crop, both terpenes and cannabinoids are predominantly synthesized in glandular trichomes, which are abundant in leaves and inflorescences but nearly absent during the early seedling stage [38].

Regarding fat-soluble phytochemicals, the present study provides the first evidence of the presence of phytosterols, tocopherols, and carotenoids in hemp sprouts. Phytosterols have been predominantly investigated in hemp seeds, where β-sitosterol was the predominant compound [39]. The results of the present study indicate that phytosterols represent the most abundant class of bioactive compounds in hemp sprouts and that both their content and composition are comparable to those previously reported in seeds (on average 1138 vs. 1240 μg g^−1^ DW), suggesting that they are largely maintained during germination. This is likely related to the crucial role that this class of compounds plays in the structure and function of cell membranes, particularly during rapid cell division and membrane biogenesis [40].

Tocopherols in hemp sprouts are mainly represented by α- and γ-tocopherol. Indeed, although β- and γ-tocopherol were not chromatographically resolved, the β+γ-tocopherol levels are largely attributable to γ-tocopherol, since β-tocopherol occurs only in trace amounts in hemp tissues [16]. Therefore, the observed profile is intermediate between those of hemp seeds, which are γ-tocopherol-dominant [41], and mature vegetative tissues, which are enriched in α-tocopherol [23], suggesting partial conversion of seed-derived γ-tocopherol into α-tocopherol by γ-tocopherol methyltransferase during germination, as also reported in other species [42]. The concurrent presence of high α- and γ-tocopherol levels may enhance protection against lipid peroxidation during early seedling growth, whereas α-tocopherol may serve to protect the photosynthetic apparatus during the de-etiolation process [43]. Lutein and violaxanthin, which represent the predominant carotenoids in hemp sprouts, may provide additional protection due to their involvement in etioplast development, de-etiolation, and photoprotection after light exposure [44].

### 4.3. Effect of Genotype on Sprout Phytochemical Content and Implications for Functional Food Applications

Beyond growth-related traits, the most relevant genotypic differences emerged at the phytochemical level. Interestingly, the different genotypes exhibited distinct patterns of metabolite accumulation during germination. Fibrante, Futura 75, and Santhica 27 generally displayed relatively higher levels across several phytochemical classes, whereas CS, Codimono, USO 31, and Felsinea showed more selective enrichment patterns. Carmaleonte was characterized by comparatively low levels across most of the metabolites evaluated. Overall, the broader distribution of phytochemicals observed in Fibrante, Futura 75, and Santhica 27 identifies these genotypes as promising candidates for further investigation in functional food applications. In particular, these genotypes showed the highest levels of phenolic compounds, with flavonoids ranging from 700 to 970 μg g^−1^ DW, corresponding approximately to 9.7–14.4 mg 100 g^−1^ FW. Although differences in plant species and tissues, extraction procedures, and analytical methodologies limit direct quantitative comparisons among studies, these values fall within, or in some cases exceed, the range typically reported for many fruits and vegetables [45]. Flavonoids are well known for their antioxidant properties and have shown multiple bioactivities in in vitro and animal studies, while epidemiological studies have associated higher flavonoid intake with a lower risk of chronic diseases [13]. In addition, Fibrante, Futura 75, and Santhica 27 exhibited high tocopherol levels, with α-tocopherol and γ-tocopherol reaching approximately 130 and 300 μg g^−1^ DW, respectively, while Fibrante showed the highest lutein level, approximately 50 μg g^−1^ DW. When expressed on a fresh weight basis, these correspond approximately to 1.8, 4.3 and 0.75 mg 100 g^−1^ FW, respectively, values comparable to or even higher than those reported for various common edible plants [46,47]. Tocopherols protect membranes from oxidative damage and have been investigated for their potential role in health promotion and chronic disease prevention [48], while lutein is a dietary carotenoid recognized for its role in eye health and, more recently, studied for its possible benefits to brain health [49,50]. The high total antioxidant activity observed in the sprouts of Fibrante, Futura 75, and Santhica 27 was associated with the accumulation of phenolic compounds, several of which showed a positive correlation with both radical scavenging activity and reducing power. Although their contribution to antioxidant activity was not directly assessed, it is feasible that the high accumulation of tocopherols and carotenoids, which also characterizes these genotypes, may contribute to the overall antioxidant potential of hemp sprouts by providing complementary antioxidant defenses of both a hydrophilic and lipophilic nature. This balanced antioxidant matrix is important from a nutritional point of view since the coexistence of structurally different redox-active phytochemicals may provide complementary and, in some cases, synergistic effects within the food matrix [51], although the relative contribution of each phytochemical class cannot be determined from the present data. In addition, the presence of significant amounts of phytosterols could further increase the nutritional interest of these sprouts, thanks to their established LDL-cholesterol-lowering properties when consumed in adequate amounts [40].

Overall, these findings indicate that, among the genotypes investigated, Fibrante, Futura 75, and Santhica 27 are the most promising in terms of nutritional quality, thanks to their greater accumulation of bioactive compounds and higher antioxidant activity. Among these, Fibrante and Santhica 27 combined superior nutritional value with high biomass production; notably, Fibrante demonstrated the best agronomic performance and the broadest phytochemical enrichment. The absence of detectable cannabinoids further supports the potential use of these sprouts in food applications from both consumer safety and regulatory perspectives. Therefore, these genotypes may represent promising candidates for sprout production and food industry applications, either for direct consumption or incorporation into food formulations, and warrant further investigation for the development of value-added plant-based foods.

## 5. Conclusions

The present study provides a comprehensive multi-class characterization of the phytochemical profile of hemp sprouts across different genotypes, highlighting the important role that genetic background plays in influencing the accumulation of bioactive compounds and antioxidant activity during the early stages of development. Significant genotypic variability was observed in biomass production and, more significantly, in the qualitative and quantitative phytochemical composition. These findings demonstrate that genotype selection is an effective strategy to improve the phytochemical composition and antioxidant properties of hemp sprouts, which may have implications for their nutritional and functional properties. The identification of hemp genotypes with specific phytochemical signatures is useful for tailored exploitation in functional food applications. Future studies are needed to clarify the role of genotype-by-environment interaction and optimize cultivation and postharvest handling strategies to maximize the phytochemical accumulation and functional properties of hemp sprouts. Furthermore, microbiological safety, shelf life, sensory acceptability, and stability and bioaccessibility of bioactive compounds during storage and processing should be investigated before the large-scale commercial exploitation of hemp sprouts as functional foods.

## Figures and Tables

**Table 1:** Sprout length and biomass accumulation across the eight genotypes investigated.

Genotype	Length (cm)	FW (mg)	DW (mg)	DW/FW
CS	7.7 ab	80.1 ab	12.3 ab	0.15
Fibrante	8.0 a	80.8 ab	11.2 abc	0.14
Carmaleonte	6.6 b	49.7 d	7.4 d	0.15
Codimono	7.0 ab	55.7 cd	8.9 cd	0.16
Futura 75	7.4 ab	69.9 abc	10.4 abc	0.15
USO 31	7.7 ab	67.1 bcd	10.0 bcd	0.15
Felsinea	7.0 ab	82.2 ab	12.0 ab	0.15
Santhica 27	7.3 ab	89.5 a	12.7 a	0.14

For each trait, different lower-case letters represent significant differences among genotypes according to Tukey’s HSD test (*p* < 0.05).

**Table 2:** Sprout terpene content across the eight genotypes investigated.

Genotype	β-Caryophyllene	α-Humulene	β-Guaiene	δ-Cadinene	Alloaromaden Drene Oxide	α-Bisabolol
CS	0.40 a	0.27 c	0.15 a	n.d.	0.36 b	0.23 b
Fibrante	0.31 bc	0.30 bc	n.d.	n.d.	0.47 b	n.d.
Carmaleonte	0.32 bc	0.32 bc	0.19 a	n.d.	0.35 b	0.32 b
Codimono	0.25 d	0.35 b	0.08 b	n.d.	0.38 b	0.33 b
Futura 75	0.33 bc	0.33 bc	n.d.	n.d.	n.d.	n.d.
USO 31	0.27 cd	0.35 b	n.d.	0.19	0.40 b	0.34 b
Felsinea	0.37 ab	0.44 a	n.d.	n.d.	0.73 a	0.66 a
Santhica 27	0.31 bc	0.27 c	n.d.	n.d.	n.d.	n.d.

Terpene content was expressed as μg g^−1^ of dry weight. n.d., not detected. For each compound, different lower-case letters represent significant differences among treatments according to Tukey’s HSD test (*p* < 0.05).

**Table 3:** Sprout total antioxidant activity across the eight genotypes investigated.

Genotype	ABTS	FRAP
CS	8.66 ab	15.83 ab
Fibrante	10.49 a	18.51 a
Carmaleonte	8.15 b	13.90 b
Codimono	8.75 ab	13.86 b
Futura 75	9.76 ab	16.71 ab
USO 31	8.51 b	14.72 b
Felsinea	8.28 b	16.60 ab
Santhica 27	9.41 ab	16.53 ab

In both the assays, the total antioxidant activity was expressed as μmol Trolox g^−1^ DW. For each assay, different lower-case letters represent significant differences among treatments according to Tukey’s HSD test (*p* < 0.05).

## Data Availability

The original contributions presented in the study are included in the article; further inquiries can be directed to the corresponding author.
